# Trends of malaria cases (plasmodium species) in Gute Health Center, Wayu Tuka District, East Wollega Zone, (2013–2022): A cross‐sectional study

**DOI:** 10.1002/hsr2.2156

**Published:** 2024-05-23

**Authors:** Temesgen Tafesse, Ra'el Desalegn, Alemayehu Dereje, Chimdesa Tolera, Dejene Desalegn, Desalegn Amenu

**Affiliations:** ^1^ Microbiology and Microbial‐biotechnology Armauer Hansen Research Institute Addis Ababa Ethiopia; ^2^ Public Health East Wollega Zonal Health Nekemte Ethiopia; ^3^ College of Natural and Computational Science, Department of Biology Wollega University Nekemte Ethiopia

**Keywords:** malaria, malaria prevalence, plasmodium species, retrospective study

## Abstract

**Background:**

Malaria is one of the biggest public health challenges in Ethiopia that has hampered the country's economic growth and development, and the government is on track to reduce malaria prevalence by 80% by 2025.

**Objective:**

As a result, the purpose of this study was to examine the trends in malaria prevalence in Wayu Tuqa District, Gute Health Center, over the last 10 years (2013–2022).

**Material and Methods:**

A retrospective analysis was undertaken to identify the patterns of malaria cases in Wayu Tuqa District, Gute Health Center, from 2013 to 2022 by evaluating the malaria registration laboratory logbook. All socio‐demographic data, as well as the year, month, and malaria prevalence, were obtained using a predesigned data collection form from previous years.

**Results:**

In this study, 3402 (22.50%) of the total 15,040 probable patients had malaria. *P. falciparum* was the most common species, accounting for 82.84% (2818) of the total, followed by *P. vivax* (16.00%) (547). Males and people over the age of 15 were the most affected demographics.

**Conclusion:**

In this study, the highest number of malaria cases were observed in 2021 and 2022, respectively. Furthermore, the autumn season had the highest incidence of malaria cases, 40% (1339), while the spring season had the lowest prevalence, 16% (546). The general trend of plasmodium species at Gute Health Center over the previous 10 years (2013–2022) has not shown inconsistent trends. As a result, proper malaria prevention and control planning, implementation, and monitoring should be strengthened at all levels.

## 1 INTRODUCTION

Malaria remains a serious public health concern around the world, contributing significantly to sickness and mortality.^1^, ^2^ According to recent WHO reports, an estimated 219 and 241 million cases of malaria were reported in 2017 and 2020, respectively. This implies that malaria cases and deaths in 2019 and 2020 increased as compared to 2017 and 2018 reports. In line with these reports, the overall burden of malaria cases and deaths was evenlyy distributed in Africa and *P. falciparum* took the lionshare, saccountingdford more than 99.00% cases.^2^, ^3^, ^4^

Despite several efforts to minimize the mortality and morbidity rates related with malaria cases, the disease remains a major problem across Africa, notably in Ethiopia.^3^ In Ethiopia, more than 65% of the land mass of the country (seasonal variations and rain fall) make the country more endemic to the malaria and put a significant proportion of the community at the risk of malaria diseases.^5^ Currently, Ethiopia is working on the WHO Global Technical Strategy, which was set to eliminate malaria cases by 2030 by stepping up current malaria control efforts. Furthermore, the country is currently on track to reach the 2030 milestone by halving the prevalence of malaria.^2^ However, as the evidence stated by some authors revealed, malaria prevention and control strategies are becoming ineffective due to some challenges, like the development of pesticide resistance, especially chloroquine resistance plasmodium species, which make it challenging to eradicate and control malaria in Ethiopia and throughout the world.^3^, ^6^

According to Wayu Tuqa District Health center (Gute Health Center), the district health center was found to have higher malaria prevalence according to East Wollega Zone health center.^7^ To develop and implement an efficient malaria control strategy in the investigated area, it is currently necessary to investigate the trends of malaria. Therefore, the purpose of this study was to look at the trends in malaria prevalence over the past 10 years, from 2013 to 2022, in the Wayu Tuqa District, Gute Health Center.

The study revealed that Wayu Tuqa district health center has a higher malaria prevalence, as per clinical data from East Wollega Zone Health Center. Further analysis indicated that the majority of malaria cases in Wayu Tuqa district were caused by Plasmodium falciparum, highlighting the need for targeted interventions in this area.^7^ In addition, the recent data on retrospective analysis of malria conducted in Leka Dullecha showed that, there is high numbers of malria cases as East Wollega Zone in general.^8^, ^9^ To develop and implement an efficient malaria control strategy in the investigated area, it is currently necessary to investigate the trends of malaria. Therefore, the purpose of this study was to look at the trends in malaria prevalence over the past 10 years, from 2013 to 2022, in the Wayu Tuqa District, Gute Health Center.

## 2 METHODS

### 2.1 Study area and study population

**Figure 1:**
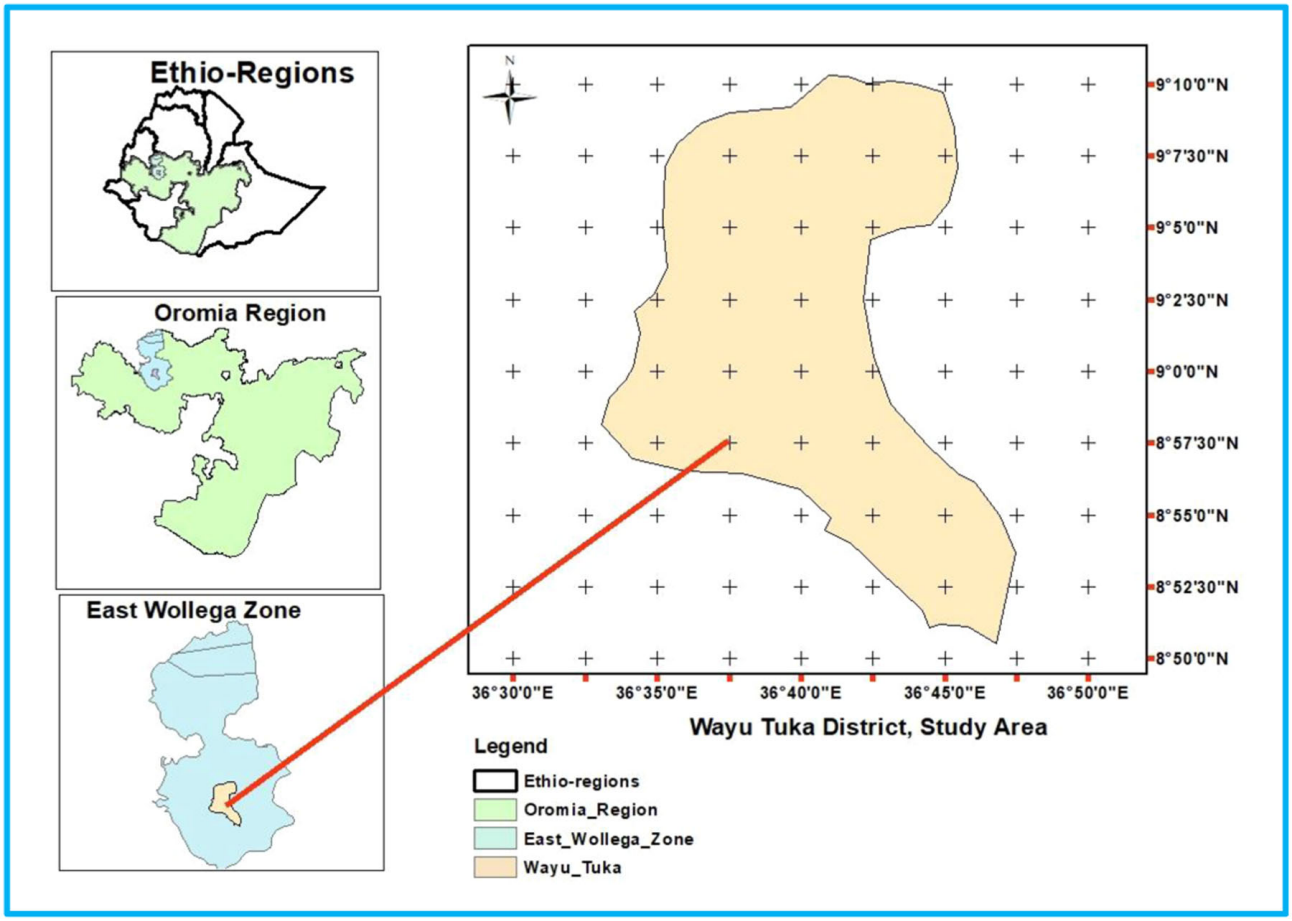
Map of the study area, East Wollega Zone, Wayu Tuka District, 2022.

The study was conducted at East Wollega Zone, Wayu Tuqa District, a public health facilty, Gute Health Center. There are 10 rural and 2 urban administrative kebeles in Wayu Tuqa Distrcit. The total population of woreda based on the 2014 population projection is **75,412** with 70325 (94.00%) in the rural, directly living on agriculture and associated activities also by supplying its produce to the neighboring urban dweller^7^ (Figure 1).

The district contains a variety of physical features, and the study area's altitude spans from 1300 to 3140 meters above sea level, according to the Wayu Tuka District Agricultural Office. A little over 17,950.8445 ha (62%) of the land area is plain, 4,922.00575 ha (17%) is hilly, and 3,763.88675 ha (13%) and 2,316.238 ha (8%), respectively, are made up of mountains and clifts. Locally referred to as “Ganna,” the rainy season lasts from May through August, peaking between June and August. The hottest months are March through October. The highest average monthly rainfall was recorded in June (4026.7 mm), and the lowest in January (99.9 mm). The coldest months of the year are from November to January, with December and January having the lowest recorded temperatures (12.2°C). The maximum mean temperature was recorded in February and March (27.9°C). The district's average annual temperature is 18.8°C, and its average annual precipitation is 2,067 mm^9^.

### 2.2 Study type and place

In this study a retrospective study from 2013 to 2022 were conducted to evaluate the trends and prevalence of *Plasmodium falciparium* in East Wollega Zone, Wayu Tuqa Distrcit, Gute Health Center (Logbook registered malaria).

### 2.3 Study period

The study period was conducted from January 2013 to December 2022.

### 2.4 Study design

To investigate the tends of malaria prevalence at Gute Health Center, a retrospective study was employed by reviewing a malaria registration logbook from 2013 to 2022.

### 2.5 Inclusion and exclusion criteria

All patients registered in logbooks recorded at the health center were included, and malaria diagnosis results that were not properly registered were excluded.

### 2.6 Sample size

In this study, totally three health facilities and 15,104 patients from logbooks were used and described as the total sample size.

### 2.7 Source of information

The malaria laboratory registered logbook (available at Gute Health Center) was used as the source of information in this investigation.

### 2.8 Malaria trend analysis

To investigate the trend of malaria prevalence over the last 10 years, laboratory registered logbook includes, malaria diagnosis date, patients gender and age, diagnosis results and parasites species used as the identification items.

### 2.9 Data quality control

Before the study began, data collectors and supervisors were trained to ensure data quality control. To ensure accuracy and consistency, the principle investigators reviewed the entire process on a daily basis, including data collection and entry.

### 2.10 Data collection and data analysis

A ten‐year period of malaria data was retrieved from a laboratory logbook using a data collection sheet, which included the year, month, sex, age, residence, total number of blood films inspected, and species type (*P. falciparum, P. vivax*, and mixed infections), and analyzed using SPSS version 26 software. Descriptive statistics were used to indicate patterns in malaria transmission by taking into account factors such as seasons, years, gender, age, and species of malaria parasite. The data was presented using figures, tables, and percentages. A statistically significant *p*‐value was defined as one that was less than 0.05. The connection of *Plasmodium* species with age group, sex, and residence was determined using mean analysis. The general pattern of malaria prevalence and malaria species distribution with habitation and season was depicted using graphs.

### 2.11 Operation definition

Malaria prevalence is defined as the total number of confirmed casesover a given period of time divided by the total number of people at risk during that same period.

A diagnostic test (quick diagnostic test and microscopy) has verified a case of malaria.

Slide positivity rate: the percentage of analyzed RDT and microscopy slides that were found to be positive.

Population at risk: people who reside in an area where there have been cases of locally acquired malaria in the past year or in the current year.

Annual parasite incidence, or API. The total number of positive slides for the malaria parasite in a year multiplied by 1000 for each population at risk is known.

### 2.12 Ethical consideration

An ethical clearance letter was obtained from the Gute Health center and Wayu Tuqa Distrcit, sent to the Administrative Office of Governmental Health Facilities in the Wayu Tuqa District, along with the reference number (EWZ‐WU‐GH/500/2022), before the commencement of data collection and pretest. When data was being collected, research participants were assured that the information would be kept private. The study received ethical approval from Aramuer Hansen Research Institute and Gute Health center and participants, obtained written consent, and conducted research in accordance with the approved Ethical consideration.

## 3 RESULTS

### 3.1 Annual trends of malaria cases at gute health center

In the past ten years (2013–2022), a total of **15,104** patients have been diagnosed for malaria cases and about **3402 (22.50%)** cases have been verified by blood tests. The number of malaria cases has fluctuated over the past 10 years, with the lowest number (**84 or 2.50%)** being reported in 2017 and the highest number (**735** or **21.55%**) being reported in **2021** (Table 1).

**Table 1:** Distribution of plasmodium species in sex and ages at Gute Health Center (2013–2022).

Year	Total Examined	*P. falcipariuum*			*P. vivax*			Mixed		
		Male	Female	Total	Male	Female	Total	Male	Female	Total
2013	826	127	78	205	34	12	46	1	0	1
2014	931	145	70	215	15	5	20	0	0	0
2015	1300	187	90	277	22	9	31	0	0	0
2016	1203	178	77	255	14	6	20	0	0	0
2017	1400	33	23	56	18	10	28	0	0	0
2018	1000	104	52	156	23	20	43	7	3	10
2019	2154	114	79	193	39	21	60	8	0	8
2020	1949	207	135	342	47	37	84	6	0	6
2021	2243	384	244	628	65	36	101	6	0	6
2022	2098	327	164	491	69	45	114	3	3	6
Total	**15,104.00**	1,806	1012	**2,818**	346	201	**547**	31	6	**37**

**Figure 2:**
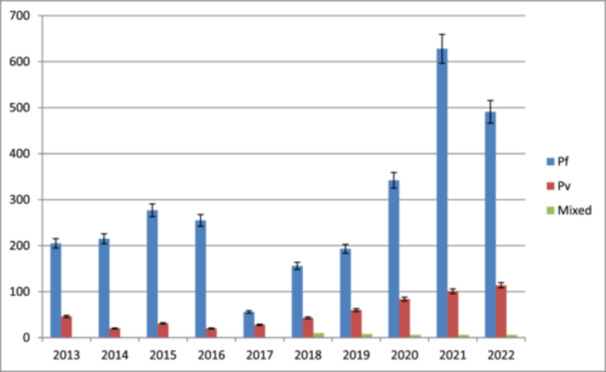
Distribution of plasmodium species composition in last ten years (2013–2022) at Gute Health Center, East Wollega Zone.

*P. falciparum* and *P. vivax* cases reported at Gute Health center for the last 10 years (2013‐2022), both plasmodium species were reported with the consultative 10 years. Hence, from the total confirmed cases, *P falciparum* and *P. vivax*, were reported as the most dominant parasites, **2818** (82.60%) and **547** (16.00%), respectively (Figure 2).

### 3.2 Ten years (2013–2022) trend analysis of malaria

**Figure 3:**
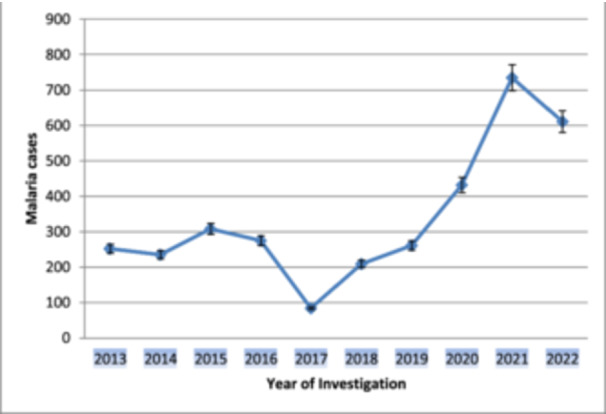
Trends of malaria cases at Gute Health center from 2013 to 2022.

We examined trend analysis of malaria prevalence over the past decade by referencing laboratory diaries registered from 2013 to 2022. Of these, 3,402 patients were recorded as malaria‐positive. This represents approximately 22.50% of the Good Health Center's malaria prevalence. Year‐over‐year malaria cases showed a gradual decline in 2016–2017, with the highest number of malaria cases in 2020–2022 (Figure 3).

### 3.3 Prevalence of malaria with respect to sex and age groups

Males were more impacted by malaria parasites than females in the research area over the last 10 years (2013–2022), according to an examination of records. There were 2287 men and 1309 females among the 3402 malaria‐positive people. The age group greater than 15 years was the most vulnerable, with an overall prevalence of (52.44%) (Table 2).

**Table 2:** Prevalence of plasmodium species with respect to sex and age in Gute Health center, 2013–2022.

Parasites	Sex	Ages		
		>5	<6–15>	>15
*P. falciparium*	Male	89	819	970
	Female	54	475	588
*P. vivax*	Male	25	146	208
	Female	13	74	103
Mixed	Male	4	11	15
	Female	0	0	2
Total		**185**	**1525**	**1886**

### 3.4 Seasonal variation of malaria prevalence in Gute Health Center from 2013 to 2022

Throughout the past 10 years, there has been a change in the monthly and seasonal distribution of malaria and infected cases (Table 3). The months of February through April had the lowest number of malaria cases (20.00%), whereas the biggest peak of malaria prevalence was recorded from September to December, which accounted for 48.50% shortly after the heavy rain season (Table 3).

**Table 3:** Seosonal distribution of plasmodium species at Gute Health Center from 2013 to 2022.

Months	Total Positive	*P. falciparium*	*P. vivax*	Mixed
September	397	340	54	3
October	439	359	73	7
November	503	421	78	4
December	405	338	66	1
January	374	304	66	0
February	148	128	18	2
March	184	152	29	3
April	168	145	23	0
May	194	164	27	3
June	192	151	37	4
July	294	244	40	0
August	298	248	48	2
**Total**	**3596**	**2994**	**559**	**29**

## 4 DISCUSSION

In this study, a total of **3402 (22.50%)** malaria cases were reported at Gute Health Center between 2013 and 2022; hence, the overall prevalence of malaria was **22.50%**. The current study reported the highest and lowest prevalence values in 2021 and 2017, respectively. Generally, the current finding is lower than the studies conducted at Asossa Hospital^10^ and Walga Health Center,^11^ which reported the malaria prevalence at 53.68% and 33.80%, respectively. Similarly, the same results were reported at Leqa Dullecha Health Center, which had about 24.4% over the overall prevalence rate.^8^ This result, however, was more than that of a study carried out at Metema Hospital^12^ and Motta Health Center,^13^ where the authors indicated that the general prevalence of malaria accounted for 17% and 11.53% of cases, respectively. This discrepancy could be explained by variations in the sample size, environment, and altitude. It is important to consider these factors when interpreting the results of the study. Additionally, future research could explore how these variables impact the findings further. Furthermore, malaria prevalence patterns did not reflect proportional trends, with 2019 and 2021 indicating the biggest peak for malaria cases, because these cases were more associated with a lack of adequate treatment and an overload of COVID‐19 characteristically over the previous 3 years.

In this study, the most impacted age groups in terms of malaria prevalence were those over the age of 15, followed by those aged 6–15, and children under the age of 5. This finding is similar to earlier studies conducted in different places, which indicated a higher prevalence of malaria in 15‐year‐olds.^14^, ^15^ In this study, a retrospective analysis found males were almost twice as likely as females to contract *Plasmodium falciparium*, which aligns with the overall gender distribution of malaria cases. This finding is consistent with previous studies,^1^, ^8^, ^13^, ^14^, ^16^ where authors also observed a higher rate of malaria cases among males compared to females. This could be due to the fact that males are more likely to get malaria since they engage in outside activities such as agricultural and pastoral work, as well as lingering outside during exophagic mosquito biting hours.

According to the prevalence of plasmodium species showed that, *P. falciparum* (83.20%), *P. vivax* (16.00%), and mixed infection (1%) were the most prevalent plasmodium species found. This finding is congruent with the findings of research conducted at Metema Hospital, which found that *P. falciparum* accounted for 90.7% of cases, *P. vivax* accounted for 9%, and mixed infection accounted for 0.3%.^13^ This research, however, conflicts with a study that was undertaken in the Jimma zone of the Assendabo Health Center, where *P. vivax* and *P. falciparum* prevalence rates were 45.75% and 54.3%, respectively.^17^ This data is consistent with the distribution of Plasmodium spp. described in various Ethiopian locations.^6^, ^8^, ^14^, ^18^ The most common parasite found was *P. falciparum*, which fluctuated over the course of the study. *P. falciparum's* dominance over *P. vivax* may be explained by the fact that it multiplies quickly, infects all ages of red blood cells, and is resistant to antimalarial drugs.

## 5 LIMITATION OF THE STUDY

One of the study's shortcomings is a lack of qualitative data on malaria intervention and control efforts during a ten‐year period, as well as a lack of case fatality rate and clinical condition data, as well as an incomplete laboratory registration book.

## 6 CONCLUSIONS

The trend analysis in this study could not fully reveal all of the malaria trends in the study area because some people seeking malaria diagnosis and treatment prefer to go to local private clinics, health posts, and drug stores, making conclusions based solely on records of patients served at the selected health facilities difficult. Furthermore, microscopy and RDTs were used in this study, which are known for their poor performance when compared to advanced molecular techniques such as polymerase chain reaction and loop‐mediated isothermal amplification, which produce highly sensitive and specific results.

The malaria trend over the last 10 years has demonstrated no regular pattern of cases in different years. Malaria transmission patterns have shown interannual and intra‐annual fluctuations, as have inconsistencies in illness burden distribution across age groups and gender. Understanding the spread of disease in time and place is essential for efficient intervention planning.

## AUTHOR CONTRIBUTIONS

**Temesgen Tafesse**: Conceptualization; Investigation; funding acquisition; writing–original draft; writing–review and editing; visualization; validation; methodology; software; formal analysis; project administration; data curation; resources. **Ra'el Desalegn**: Conceptualization; investigation; funding acquisition; writing–original draft; methodology; visualization; validation; project administration; formal analysis; writing–review and editing; software; data curation; supervision; resources. **Alemayehu Dereje**: Conceptualization; investigation; funding acquisition; writing–original draft; writing–review and editing; visualization; validation; methodology; software; formal analysis; project administration; data curation; supervision; resources. **Chimdesa Tolera**: Data curation; supervision; resources; project administration; formal analysis; software; methodology; validation; visualization; writing–review and editing; writing–original draft; funding acquisition; investigation; conceptualization. **Dejene Desalegn**: Investigation; conceptualization; funding acquisition; writing–original draft; methodology; validation; visualization; writing–review and editing; software; formal analysis; project administration; data curation; supervision; resources. **Desalegn Amenu**: Data curation; supervision; resources; project administration; formal analysis; software; methodology; validation; visualization; writing–review and editing; writing–original draft; funding acquisition; investigation; conceptualization.

## CONFLICT OF INTEREST STATEMENT

The authors declare no conflicts of interest.

## TRANSPARENCY STATEMENT

The lead author Desalegn Amenu affirms that this manuscript is an honest, accurate, and transparent account of the study being reported; that no important aspects of the study have been omitted; and that any discrepancies from the study as planned (and, if relevant, registered) have been explained.

## ETHICS STATEMENT

An ethical clearance letter was obtained from the Gute Health center and Wayu Tuqa Distrcit, sent to the Administrative Office of Governmental Health Facilities in the Wayu Tuqa District, along with the reference number (EWZ‐WU‐GH/500/2022), before the commencement of data collection and pretest. When data was being collected, research participants were assured that the information would be kept private.

## Data Availability

The data that support the findings of this study are available on request from the corresponding author. The data are not publicly available due to privacy or ethical restrictions.
